# How cancer cells dictate their microenvironment: present roles of extracellular vesicles

**DOI:** 10.1007/s00018-016-2346-3

**Published:** 2016-08-31

**Authors:** Yutaka Naito, Yusuke Yoshioka, Yusuke Yamamoto, Takahiro Ochiya

**Affiliations:** Division of Molecular and Cellular Medicine, National Cancer Center Research Institute, 5-1-1, Tsukiji, Chuo-ku, Tokyo, 104-0045 Japan

**Keywords:** Exosome, Microvesicle, Apoptotic body, Fibroblasts, Immune cells, Endothelial cells, Epithelial cells, Mesenchymal stem cells

## Abstract

Intercellular communication plays an important role in cancer initiation and progression through secretory molecules, including growth factors and cytokines. Recent advances have revealed that small membrane vesicles, termed extracellular vesicles (EVs), served as a regulatory agent in the intercellular communication of cancer. EVs enable the transfer of functional molecules, including proteins, mRNA and microRNAs (miRNAs), into recipient cells. Cancer cells utilize EVs to dictate the unique phenotype of surrounding cells, thereby promoting cancer progression. Against such “education” by cancer cells, non-tumoral cells suppress cancer initiation and progression via EVs. Therefore, researchers consider EVs to be important cues to clarify the molecular mechanisms of cancer biology. Understanding the functions of EVs in cancer progression is an important aspect of cancer biology that has not been previously elucidated. In this review, we summarize experimental data that indicate the pivotal roles of EVs in cancer progression.

## Introduction

The malignant phenotypes of tumors not only are determined by cancer cells themselves but also depend on their surrounding tumor microenvironments [1, 2]. These microenvironments consist of various cell types, such as fibroblasts, lymphocyte, inflammatory cells, epithelial cells, endothelial cells, and mesenchymal stem cells. These cells within the tumor microenvironment and cancer cells interact with each other and form the intrinsic communication networks that affect several cancer hallmarks, as described by Hanahan and Weinberg [3]. Several reports documented that such intercellular communications were modulated by various humoral factors, such as growth factors, cytokines, and chemokines. Similar to these molecules, recent advances in cancer biology revealed that extracellular vesicles (EVs) also served as a regulatory agent in such communications.

EVs have a heterogenetic population and are generally categorized as exosome, microvesicles or ectosomes, and apoptotic bodies [4–6]. These vesicles originate from different subcellular compartments [4–6]. Exosomes are small membrane vesicles, ranging from 50 to 150 nm in diameter, that have a lipid bilayer membrane and originate from the exocytosis of multivesicular bodies (MVBs) containing intraluminal vesicles [6]. Exosome biogenesis and release are modulated by the endosomal sorting complex that is required for transport (ESCRT) machinery and the ceramide-dependent pathway [6]. Researchers in EV biology have identified several types of exosome markers, including tetraspanins (CD9, CD63, CD81), heat shock proteins (HSP60, 70, and 90), membrane transporters and fusion proteins (Annexins and flotillin), and MVB synthesis proteins (Alix and TSG101) [7]. Microvesicles are 100–1000 nm in diameter and are produced directly from the plasma membrane via budding [8]. Microvesicles are enriched in some lipid components and phosphatidylserine [9]. The biogenesis of microvesicles is modulated by the interaction between phospholipid redistribution and the contraction of cytoskeletal structures [10]. Apoptotic bodies (500–4000 nm in diameter) are formed during the apoptotic process and contain organelles and nuclear fragments [6, 10, 11]. Apoptotic bodies also contain DNA fragments and RNA. Macrophages subsequently clear apoptotic bodies by phagocytosis [11]. However, these apoptotic bodies may participate in the intercellular communication of the cancer microenvironment. Indeed, H-ras^V12^- and human c-myc-transfected to rat fibroblasts could transfer their DNA to other fibroblasts by apoptotic bodies, thereby inducing tumorigenic phenotypes [12].

**Fig. 1:**
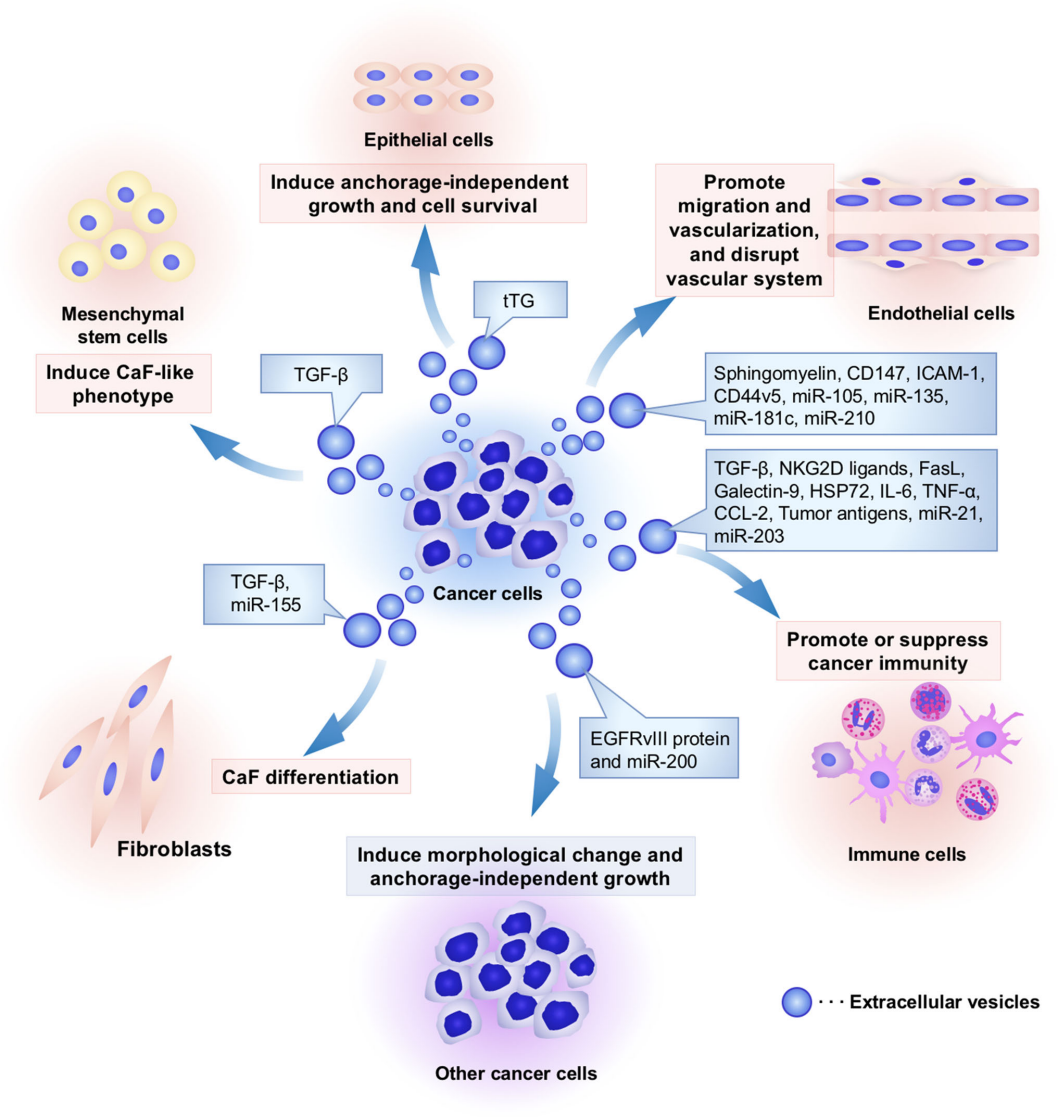
Cancer cell-derived EVs modify the characters of cancer surrounding microenvironment. Several kinds of cell types, such as cancer cells, fibroblasts, immune cells, endothelial cells, epithelial cells, and mesenchymal stem cells, comprise unique microenvironment for cancer progression. Cancer cells utilize EVs to modify surrounding cells within tumor microenvironment. Cancer-derived EVs have multiple functions that depend on component molecules of EVs. To induce cancer-associated fibroblast (CaF)-like phenotypes in cancer surrounding fibroblasts and mesenchymal stem cells, cancer cells secrete EVs and transfer growth factors and microRNAs (miRNAs), including transforming growth factor-beta (TGF-β) and miR-155, respectively. To escape from immune surveillance, cancer cells transfer several types of immunoregulatory molecules into immune cells. However, these cancer-derived EVs also stimulate cancer immunity to kill tumor cells because tumor antigens were packaged in EVs and stimulated cancer immunity. Cancer-derived EVs also contain angiogenic proteins and miRNAs that promote migration and proangiogenic activity of endothelial cells. In addition, miR-105 and miR-181c in EVs are capable of rupturing the vascular system to increase the permeability that supports cancer metastasis. Cancer-derived EVs confer malignant phenotypes in other cancer cells and epithelial cells by transferring oncogenic proteins and miRNAs, such as EGFRvIII, miR-200, and tissue transglutaminase (tTG). Taken together, cancer cells “dictate” the characters of their surrounding stromal cells and create a convenient microenvironment to support cancer progression via EVs

**Fig. 2:**
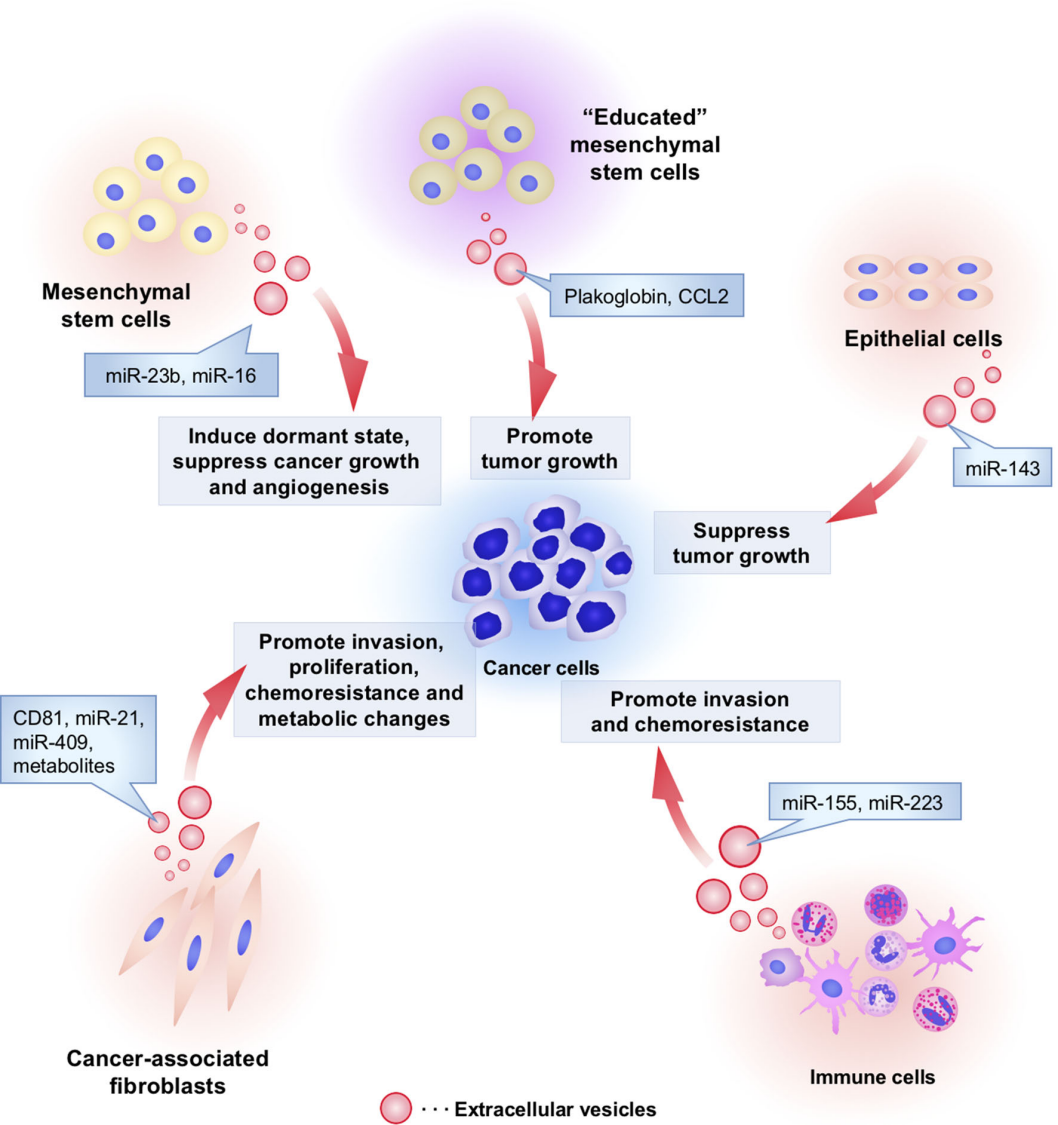
The functional role of non-tumoral cell-derived EVs in cancer initiation and progression. Non-tumoral cells utilize EVs to affect cancer initiation and progression. Cancer-associated fibroblasts secrete EVs and affect invasion, proliferation, chemoresistance, and metabolic properties of cancer cells by transferring CD81, miR-21, miR-409, and metabolites. Macrophage-derived EVs contain miR-223, which stimulates the invasive activity of cancer cells. Monocytes transfer miR-155 to neuroblastoma via EVs and induce chemoresistance in neuroblastoma. “Non-educated” mesenchymal stem cells (MSCs) by cancer cells secrete EVs containing miR-16 to suppress tumor growth and angiogenesis. However, interestingly, “educated” MSCs by cancer cells enable to secrete EVs that contain tumor promotive proteins such as plakoglobin and CCL2. MSC-derived EVs also transfer miR-23b, which induce dormant state of cancer cells to survive in a quiescent state while waiting for the appropriate environmental conditions to begin proliferation again. Non-aberrant epithelial cells secreted EVs to transfer miR-143 into cancer cells and suppress tumor growth

EVs contain functional cellular components such as proteins, mRNAs, and microRNAs (miRNAs) that enable the transfer of these principal factors to various cell types [13]. These components of EVs are also functional in the recipient cells and are highly variable depending on the origin cells [6]. As shown in Figs. 1 and 2, this EV-mediated interaction between cancer cells and their surrounding cells within tumor microenvironment confers advantages for cancer initiation and progression. Non-tumoral cells also utilize EVs to transfer the tumor-suppressive molecules that affect cancer initiation and progression (Fig. 2). Therefore, researchers consider EVs to be important cues for understanding the molecular mechanisms underlying the intercellular communication in the tumor microenvironment. In this review, we will summarize the current knowledge regarding the functional role of EV components on intercellular communication between cancer cells and each cell type within the tumor microenvironment.

## Interaction between cancer cells and surrounding stromal fibroblasts via EVs

The fibroblasts within tumor stroma, which are also termed cancer-associated fibroblasts (CaFs), have heterogeneous populations and include myofibroblasts that are similar to fibroblasts associated with wound healing [14]. CaFs enable the formation of a unique microenvironment that plays a pivotal role in cancer development and progression. Although the origins of CaFs and the signaling that mediates CaF induction remain controversial, several types of factors, including transforming growth factor-beta (TGF-β), are required for the induction and maintenance of CaFs [14–18]. In addition to these factors, the EVs derived from cancer cells induce such CaF-like phenotypes in cancer surrounding stromal cells (Table 1). Webber et al. showed that TGF-β was loaded on the surface of EVs derived from prostate cancer and mesothelioma cell lines [19, 20]. These cancer-derived EVs could trigger the TGF-β/SMAD3 signaling pathway in fibroblasts and induce the myofibroblast-like phenotypes, including the induction of α-smooth muscle action (α-SMA) expression and the production of fibroblasts growth factor 2 (FGF2) [19]. These data suggest that cancer cells could dictate the characters of their surrounding stromal cells via EVs and create a convenient microenvironment to support cancer cell survival and progression.

**Table 1:** EV interaction between cancer cells and fibroblasts

Cell types of EV donor	Cell types of EV recipient	EV components	Functions	References
Positive regulation of extracellular vesicles on cancer progression				
Cancer cells	Fibroblasts	TGF-β	Triger the myofibroblast differentiation	[19]
		TGF-β	Triger the myofibroblast differentiation and promote cancer growth and angiogenesis	[20]
		Integrins	Up-regulates S100 gene expressions and promote cell growth and migration	[38]
		miR-155	Induce cancer-associated fibroblast-like phenotype through repressing TP53P1	[26]
		miR-122	Down-regulates glucose consumption of fibroblasts	[35]
Fibroblasts	Cancer cells	CD81	Enhance cancer motility and metastasis	[21]
		Extracellular matrix proteins and ADAM10	Promote cancer motility	[16]
		Non-coding of transposable RNAs	Expansion of therapy-resistant tumor-initiating cells	[23]
		miR-409	Promote epithelial-mesenchymal transition	[27]
		Wnt3a	Expansion of cancer stem cell to enhance chemoresistance	[39]
		miR-21 isomiR	Confer the chemo-resistance through targeting APAF1	[28]
		Metabolites including amino acids and lipids	Affect of metabolic properties of cancer cells	[33]

In contrast, stromal fibroblasts that are “educated” by cancer cells also secreted EVs and established the communication networks that provide the ultimate benefit for cancer progression. Luga et al. demonstrated that CD81-positive CaF-derived EVs enhanced breast cancer cell motility and metastasis by activating the Wnt-planar cell polarity (PCP) signaling pathway [21]. Endocytosis and internalization of the Wnt-receptor complex from the plasma membrane are required for signal transduction [22]. Wnt11 and CD81 are localized on EVs, and the internalization of CD81 molecules on the EVs in breast cancer cell may support the endocytic trafficking of autocrine Wnt ligand to activate the Wnt-PCP pathway [21]. Because CD81 is a well-known marker of EVs [7], it is possible that CD81-positive EVs derived from other cell types within the tumor microenvironment may contribute to the activation of the Wnt-PCP pathway in cancer cells. Boelens et al. demonstrated that resistance to chemotherapy and radiation in breast cancer is mediated by CaF-derived EVs [23]. These CaF-derived EVs contain non-coding or transposable RNAs that stimulate RIG-I recognition to activate STAT1. Activated STAT1 cooperates with juxtacrine-activated NOTCH3 to mediate NOTCH target gene transcriptions. NOTCH and its target genes support the maintainance of tumor-initiating cells that are known to be resistant to chemotherapy and radiation [24]. Therefore, NOTCH target gene induction via EVs results in the expansion of therapy-resistant tumor-initiating cells [23]. Interestingly, CaF-derived EVs preferentially affect breast cancer cells with the basal-like subtype that is associated with aggressive phenotype of breast cancer [23, 25]. Although the mechanism by which CaF-derived EVs could preferentially affect basal-like subtype breast cancer cells has not been addressed, CaF-derived EVs potentially have a tropism to influence treatment-resistant cancer cells.

Recently, an increasing number of reports have demonstrated that secreted miRNAs also enable to compose the communication networks between cancer cells and CaFs to modulate cancer progression. Pang et al. showed that miR-155 was secreted through cancer-derived EVs and dictated CaF-like phenotypes in fibroblasts by repressing TP53INP1 [26]. This finding suggests that cancer-derived EVs also affect stromal fibroblasts through transferring miRNAs. However, Josson et al. demonstrated that CaF-derived EVs contain miR-409, which promotes the epithelial-to-mesenchymal transition (EMT) [27]. Moreover, Yeung et al. demonstrated that CaFs and cancer-associated adipocytes secreted miR-21 via EVs and conferred chemo-resistance to cancer cells by regulating apoptotic peptidase activating factor 1 (APAF1) expression [28]. Because miRNA expression changes involve regulating the tumor-promoting function of CaFs [29], the miRNA expression profile in CaFs and their EVs reflect the disease status of cancer. Understanding EV communication networks in tumor-stromal interaction will enable the development of therapeutic strategies to target EVs and may provide novel functional marker of CaFs.

Cancer cells can reprogram their energy metabolism to be distinct from that of normal cells [3, 30]. Interestingly, CaFs within tumor stroma also support the metabolic properties of cancer cells [31, 32]. Zhao et al. showed that CaF-derived EVs alter the metabolic properties of prostate cancer cells [33]. In the presence of CaF-derived EVs, oxidative phosphorylation is inhibited, but glycolysis and lactate levels are increased in prostate cancer cell lines. This finding suggests that EVs induce the “Warburg effect” [30, 34]. These CaF-derived EVs also transfer TCA cycle metabolites, amino acids, and lipids into cancer cells. Interestingly, breast cancer-derived EVs also modify the glucose consumption of fibroblasts in metastatic sites by targeting pyruvate kinases [35]. Therefore, cancer-derived EVs enable to educate stromal cells within distant niche to promote cancer metastasis. Similarly, several reports also showed the important aspects of cancer-derived EV on the cancer progression through remodeling the stromal cells within pre-metastatic niche [36, 37]. Although further studies are required to clarify the advantages of metabolic change in cancer progression, these findings suggest that the communication networks of EVs contribute to cancer proliferation and survival in the surrounding environment with limited oxygen and nutrient supplies.

## Cancer-derived EVs in the immune system

The major function of the immune system is not only to protect against several infectious pathogens but also to eradicate the abnormal cells [40]. Therefore, to promote cancer initiation and progression, evasion from immune surveillance is one of the key hallmarks of cancer cells [3]. However, increasing evidences indicate that the immune systems play a dual role in cancer progression and can both suppress and support the tumor cells. In addition, several reports suggested that macrophages and neutrophils within tumor tissue were associated with cancer progression [40–44]. These macrophages and neutrophils also exhibit polarity on either a tumor-suppressive or a tumor-promoting phenotype, depending on the surrounding microenvironment [43, 44]. Hence, cancer cells and immune cells form intricate communication networks that affect the risk of cancer development.

EVs also modulate the evasion of cancer cells from immune surveillance (Table 2). For example, cancer-derived EVs induce apoptosis of CD8-positive T-cells and promote regulatory T-cell expansion to suppress anticancer immunity [45, 46]. Additionally, cancer-derived EVs included several types of immunoregulatory molecules, such as FasL [47], TGF-β [48], NKG2D ligands [49], galectin-9 [50], and HSP72 [51], to support the immune escape of cancer cells. These EVs also induce the differentiation of monocytes into immunosuppressive macrophages [52]. Interestingly, cancer-derived EVs increase the survival rate of monocytes [53, 54]. Consequently, cancer-derived EVs facilitate the formation of macrophages from monocytes in the tumor microenvironment. These macrophages are also capable of producing the EVs that enhance the metastatic properties of cancer cells [55]. Moreover, cancer-derived EVs internalize into macrophages and induce pro-inflammatory cytokines, including IL-6, TNF-α, and CCL2, through activating NF-κB signaling [56]. Thus, EV communications between cancer cells and immune cells seem to provide the advantage of evasion from immune surveillance and cancer progression. However, several researchers suggested that tumor antigens were packaged in EVs and stimulated cancer immunity to kill tumor cells [57, 58]. Therefore, cancer-derived EVs are also utilized as anti-tumoral vaccines [59, 60]. In addition, dendritic cells (DC)-derived EVs induce CD8-positive T-cell activation in both a CD4-positive T-cell and B-cell-dependent manner [61]. The availability of these DC-derived EVs as a tool of vaccine immunotherapy was also tested [62–64]. Taken together, EVs within the tumor microenvironment also participate in such complex interactions between cancer cells and immune cells.

**Table 2:** EV interaction between cancer cells and immune cells

Cell types of EV donor	Cell types of EV recipient	EV components	Functions	References
Positive regulation of extracellular vesicles on cancer progression				
Cancer cells	Immune cells	FasL	Induce the apoptosis of lymphocytes	[47]
		FasL and HLA class I antigens	Induce apoptosis and caspase activation	[71]
		FasL and TRAIL	Induce T-cell apoptosis	[72]
		Several proteins including chemokine receptor 6 (CCR6) and CD44 variant 7/8	Increase the survival rate of monocytes	[53]
		CD44	Induce tumor-associated macrophage-like phenotypes from monocytes	[73]
		TGF-β	Inhibit NK cell cytotoxic function and enhance regulatory T cell immune suppressive function	[48]
		NKG2D and TGF-β	Inhibit lymphocyte effector function	[49]
		Melanoma antigen (MAGE), FasL and MHC class I	Apoptosis of CD8 positive T-cell and promote regulatory T cell expansion	[45]
		Galectin-9	Induce the apoptosis of mature Th1 lymphocytes	[50]
		IL-10, TGF-β, FasL, MAGE and MHC class I	Apoptosis of CD8 positive T-cell and promote regulatory T cell expansion	[46]
		HSP72	Induce immunosuppressive activity of myeloid-derived suppressor cells	[51]
		Palmitoylated proteins	Induce pro-inflammatory cytokines, including IL-6, TNF-α and CCL2, through activating NF-κB signaling	[56]
		Chondroitin sulfate proteo-glycan 4, α2-macroglobulin, lactadherin, syntenin-1, myristoylated alanine-rich C-kinase substrate (MARCKS), integrin alpha-V, integrin alpha-3, and epithelial growth factor receptor (EGFR)	Induce the differentiation of monocyte into immunosuppressive macrophages	[52]
		Phospholylated receptor thyrosin keinase	Increase the survival rate of monocytes by regulating the MAPK pathway	[54]
		miR-203	Suppress the expression of the immune response-relative genes, and may contribute to suppressing anticancer immunity	[65]
		miR-21	Induce miR-155 expression in TLR8 dependant manner	[66]
Cancer cells	Kupffer cells in the liver	Migration inhibitory factor (MIF)	Promote TGF-β secretion in Kupffer cells and activate hepatic stellate cells to form fibrotic niche	[97]
Immune cells	Cancer cells	miR-223	Stimulates the invasive activity of breast cancer cells by regulating the Mef2c-β-catenin pathway	[55]
		miR-155	Enhance chemoresistance of cancer cells through targeting TERF1	[66]
		miR-126a	Induce IL-13 positive Th2 macrophage maturation and promote tumor angiogenesis that lead to cancer cell metastasis	[74]
Negative regulation of extracellular vesicles on cancer progression				
Cancer cells	Immune cells	Melanoma antigen (Mart1)	Stimulated cancer immunity to kill tumor cells	[57]
		Tumor antigens	Stimulated cancer immunity to kill tumor cells	[58]
Immune cells	Immune cells	Tumor peptide, MHC class I and II	Stimulated cancer immunity to kill tumor cells	[75]
		Cytosolic proteins (including hsc73) and membrane proteins (including milk fat globule-EGF-factor VIII (MFG-E8))	Stimulated cancer immunity	[76]
		MHC class I	Induce CD8-positive T-cell activation in both a CD4-positive T-cell and B-cell dependent manner	[61]

Among these communication networks of the EVs involved in cancer immunity, the miRNAs secreted via EVs are also involved in the regulation of cancer immunity (Fig. 1; Table 2). Indeed, pancreatic cancer-derived EVs containing miR-203 regulate the expression of the immune response-relative genes, including toll-like recepter-4, and may contribute to suppress anticancer immunity [65]. In addition, macrophage-derived EVs can transfer miR-223, which stimulates the invasive activity of breast cancer cells, by regulating the Mef2c-β-catenin pathway [55]. Furthermore, miR-21 is transferred into monocytes via neuroblastoma-derived EVs and can bind to TLR8 to stimulate NF-κB pathway and induce miR-155 expression in monocytes [66]. Interestingly, miR-155 is also transferred from monocytes to neuroblastoma via EVs and induces chemoresistance in neuroblastoma through targeting telomeric repeat binding factor 1 (TERF1). Such communication networks of secreted miRNAs via EVs are likely associated with programmed cell death protein ligand-1 (PD-L1) in mediating the immune response. PD-L1 is well known as a ligand of programmed cell death protein-1 (PD-1) that plays a pivotal role in T-cell inhibition and exhaustion. PD-L1 is up-regulated in several cancers to suppress the cytotoxic activity of T-cells and escape from the immune system. Instability of the PD-L1 3′-untranslated region (UTR) was recently reported to be associated with aberrant PD-L1 expression in cancer cells [67]. Importantly, most miRNA targeting sites are located in the 3′-UTR region of target genes [68]. Several reports have demonstrated the functional role of miRNAs in immune checkpoints by regulating PD-L1 expression [69, 70]. Although it is unclear that miRNAs targeting PD-L1 are packaged in EVs, it is conceivable that the genetic status of PD-L1 may affect the functional role of the secreted miRNAs that are included in EVs in the evasion of cancer cells from immune surveillance. Further understanding of the molecular mechanisms underlying these complex communication networks via EVs will provide several avenues for cancer immunotherapy and diagnosis.

## The communication network of EVs between cancer cells and endothelial cells

Angiogenesis within tumor tissue is required to provide nutrients and oxygen to cancer cells and allows entry to the blood circulation system [3, 77, 78]. Angiogenesis can trigger the formation of metastatic foci in secondary sites. Tumor vascularization is regulated by a number of potential mechanisms, and vascular endothelial cells are one of the origins of the tumor vessel [79]. Cancer-derived EVs support the vascularization by affecting endothelial cells within the tumor microenvironment and may confer the aggressive phenotypes to cancer cells (Table 3). For instance, prostate and ovarian cancer cells secrete EVs to transfer sphingomyelin and CD147 into endothelial cells. These EV molecules promote migration and proangiogenic activity of endothelial cells [80, 81]. Similarly, nasopharyngeal cancer-derived EVs contained intercellular adhesion molecule-1 (ICAM-1) and CD44 variant isoform 5 (CD44v5), which significantly increase the migration, invasion, and tubulogenesis of endothelial cells [82]. Although these reports did not clarify the contribution of these EV components in cancer progression, cancer-derived EV-induced angiogenesis within the tumor microenvironment probably supports cancer survival and metastasis.

**Table 3:** EV interaction between cancer cells and endothelial cells

Cell types of EV donor	Cell types of EV recipient	EV components	Functions	References
Positive regulation of extracellular vesicles on cancer progression				
Cancer cells	Endothelial cells	Sphingomyelin	Promote migration and vascularization of endothelial cells	[80]
		Tetraspanin CO-029/D6.1A (as known tetraspanin-8 (TSPAN-8)	Induce vascularization	[98]
		CD147	Promote migration and vascularization of endothelial cells	[81]
		Several molecules including TSPAN-8, CD49d and CD106	Enhance cell growth, migration, vascularization and maturation of endothelial cells	[99]
		IL-6, VEGF, MMP2	Induce vascularization of endothelial cells	[100]
		Intercellular adhesion molecule-1 (ICAM-1) and CD44 variant isoform 5 (CD44v5)	Promote migration and vascularization of endothelial cells	[82]
		H19 long non-coding RNA	Promote an angiogeneic phenotype in endothelial cells	[101]
		miR-9	Promote migration and vascularization of endothelial cells	[102]
		miR-210	Modulates cancer metastasis through neovascularization	[83]
		miR-210	Induce vascularization of endothelial cells	[87]
		miR-135	Induce vascularization of endothelial cells	[86]
		miR-105	Destroy the tight junction of the endothelial cells to promote metastasis	[96]
		miR-181c	Disrupt the permeability of the BBB to promote brain metastasis	[95]

In contrast, cancer cell-derived EVs also transported miRNAs into endothelial cells to modulate neovascularization. Metastatic breast cancer-derived EVs transport miR-210 into endothelial cells [83]. This miRNA secretion is regulated by neutral sphingomyelinase 2 (nSMase2), which is highly expressed in cancer cells compared with non-neoplastic cells [83–85]. Because nSMase2 expression promotes metastatic initiation, miRNA secretion via cancer-derived EVs modulates cancer metastasis through neo- vascularization. The functional role of EVs containing miR-135 and miR-210 in tumor angiogenesis is also observed in multiple myeloma and leukemia, respectively [86, 87]. Interestingly, hypoxic conditions promote the secretion of EVs containing miR-135 and miR-210 in cancer cells [86, 87]. Hypoxic conditions within tumor tissues affect several characteristics of cancer, including angiogenesis [88]. Furthermore, hypoxia promotes EV release and alteration in the EV components [89, 90]. Therefore, EVs are utilized to facilitate the malignant behavior in hypoxic tumor microenvironments. However, the exact contribution of miRNAs and proteins in EVs during angiogenesis and cancer metastasis remains unclear. Understanding these relationships will enable the development of cancer therapy. Indeed, anti-angiogenic strategies to inhibit EVs secretion and miRNA contents were examined [91].

Cancer-derived EVs mediate tumor angiogenesis within proximal microenvironments. However, cancer cells also utilize EVs for endothelial cell modifications within distant microenvironments to form a metastatic niche. For instance, the blood–brain barrier (BBB) tightly regulates the permeability from the vascular system to the central nervous system [88, 92, 93]. Thus, BBB destruction is one of the key steps to allow the extravasation of cancer cells during brain metastasis [88]. Although several types of molecules contributed to the BBB disruption [92, 94], EVs are also associated with brain metastasis of cancer cells by affecting BBB endothelial cells. A recent study indicated that brain metastasis breast cancer-derived EVs containing miR-181c were closely associated with the BBB destruction and brain metastasis [95]. miR-181c is transferred into endothelial cells of the BBB by EVs and causes the delocalization of actin by suppressing the expression of 3-phosphoinoitide-dependent protein kinase-1 (PDPK1). PDPK1 suppression leads to the degradation of the phosphorylated cofilin, which is important for actin dynamics and disrupts the permeability of the BBB. Similar to this report, Zhou et al. reported that metastatic breast cancer-derived EVs also contain miR-105 that directly inhibits tight junction protein 1 (ZO-1) expression in endothelial cells and destroys the tight junction to promote metastasis [96]. It appears that miR-181c is included in the EVs that preferentially accumulate in brain endothelial cells, but EVs that contain miR-105 may affect endothelial cells throughout the body. Therefore, cancer-derived EVs may participate in such organ-specific metastasis of cancer cells through remodeling the distal pro-metastatic niche. Indeed, pancreatic cancer-derived EVs are selectively uptaken by liver Kupffer cells and promote liver metastasis of cancer cells [97]. In addition, cancer-derived EVs had a tropism depending on surface integrin marks [38]. However, it is still controversial whether all of cancer-derived EVs exhibit specificity for the pre-metastatic niche. Clarification of these points is necessary for the use of EVs as novel therapeutic targets of cancer metastasis.

## The functional role of EVs on intercellular communication between cancer cells and mesenchymal stem cells

Cancer-derived EVs affect the mesenchymal stem cells (MSCs) as well as fibroblasts and endothelial cells (Table 4). MSCs reside in several types of mesodermal tissues, such as bone marrow (BM), adipose tissue, umbilical cord, and peripheral blood [103–105]. Several lines of evidence indicated that these MSCs are recruited into the tumor microenvironment and could promote tumor growth and metastasis [106, 107]. Cancer-derived EVs control the differentiation of BM-MSCs to a CaF-like state through activating the TGF-β signaling pathway [108]. This differentiated BM-MSC with EVs exhibits proangiogenic properties to enhance tumor proliferation and invasion. Similarly, Paggetti et al. demonstrated that chronic lymphocytic leukemia (CLL) cells also secreted EVs to induce CaF-like phenotypes in BM-MSCs and endothelial cells [109]. BM-MSCs and endothelial cells with CLL-derived EVs support CLL adhesion, survival, and growth in vitro and in vivo. The origins of CaFs are still controversial, and cancer-derived EVs also induce CaF-like phenotypes in cancer surrounding fibroblasts [14]. Therefore, cancer-derived EVs can convert several types of stromal cells into CaF-like cells. These data suggest that EVs are associated with CaF population diversity.

**Table 4:** EV interaction between cancer cells and bone marrow stromal cells

Cell types of EV donor	Cell types of EV recipient	EV components	Functions	References
Positive regulation of extracellular vesicles on cancer progression				
Cancer cells	Mesenchymal stem cells	TGF-β	Induce cancer-associated fibroblast-like phenotype through activating TGF-β/SMAD pathway	[119]
		TGF-β	Induce cancer-associated fibroblast-like phenotype and enhance tumor proliferation and invasion	[108]
		Functional miRNA and proteins	Induce cancer-associated fibroblast-like phenotype and enhance tumor proliferation	[109]
		miR-146a	Induction of several cytokines and chemokines in mesenchymal stem cells, and enhance cancer viability and migration	[120]
Cancer cells	Bone marrow progenitor cells	MET	Change the phenotype and mobilzation of bone marrow progenitor cells and support tumor angiogenesis	[121]
Mesenchymal stem cells	Cancer cells	Fibronectin, Junction plakoglobin, and CCL2 from cancer “educated” mesenchymal stem cells	Promote cancer proliferation and dissamination	[118]
		Probably transferred HGF mRNA by EVs	Promte cancer growth and migration	[116]
		miR-23b	Induce cancer cell dormancy	[113]
		miR-21, miR-34a, PDGFR-β, TIMP-1, TIMP-2, lactic acid, glutamic acid, and sphingomyelin	Increase cancer cell proliferation and survival	[122]
Bone marrow stromal cells	Cancer cells	monocyte chemo- attractant protein 1 (MCP-1), interferon-inducible protein 10 (IP-10), stromal cell-derived factor 1 (SDF-1)	Promote cancer cell growth, migration, and drug resistance	[123]
Negative regulation of extracellular vesicles on cancer progression				
Mesenchymal stem cells	Cancer cells	miR-16	Inhibit tumor vascularization through suppressing VEGF expression	[115]
		miR-15 from healthy donor-derived mesenchymal stem cells	Inhibit cancer proliferation and dissamination	[118]
Bone marrow stromal cells	Cancer cells	miR-146b	Inhibit cancer growth	[124]

Breast cancer patients have a protracted risk of recurrence within 5 years and even up to 10–20 years after surgery or adjuvant chemotherapy [110–112]. The phenomenon indicates that breast cancer cells acquire a dormant state and survive for a long time in the patient’s body. Although cancer cells cease dividing under a dormant state, they can survive in a quiescent state while waiting for the appropriate environmental conditions to begin proliferation again. Importantly, BM-MSCs contribute to the maintenance of such cancer cell dormancy via EVs [113]. BM-MSCs transfer miR-23b-containing EVs into breast cancer cells and modulate the dormant state by targeting myristoylated alanine-rich C-kinase substrate (MARCKS), which modulates cell motility and cell cycle progression [114]. Consistent with this report, it was also indicated that BM-MSCs secreted miR-16 via EVs and downregulated vascular endothelial growth factor (VEGF) expression to lead to the inhibition of growth and angiogenesis in breast cancer [115]. In contrast, several reports also showed that these MSC-derived EVs promoted tumor growth in renal cancer [116], gastric cancer, and colorectal cancer [117]. Thus, the functional role of MSC-derived EVs depends on cancer cell types. In addition, it was reported that EVs derived from BM-MSCs of multiple myeloma patients enhance tumor growth but that healthy subject-derived BM-MSCs suppress tumor growth via EVs [118]. Taken together, these different functions of MSC-derived EVs depending on the phenotypes of cancer cells. Understanding these points will provide a novel insight into cancer therapy.

## Horizontal propagation of oncogenic molecules by EVs

As mentioned above, cancer-derived EVs controlled stromal cells within the tumor microenvironment to promote cancer progression. However, cancer cells can also transfer their oncogenic properties to other cancer cells via EVs (Table 5). For instance, the epithelial growth factor receptor (EGFR) truncated mutant, EGFR variant III (EGFRvIII), is closely associated with cancer progression and poor patient prognosis of glioblastoma (GBM), which is most common brain malignancy in adults [125]. Al-Nedwi et al. reported that the EGFRvIII could be transferred from EGFRvIII-positive GBM cells to negative GBM cells through EVs [126]. Internalization of EGFRvIII-containing EVs activated the MAPK and Akt signaling pathways in the EGFRvIII-negative GBM cells, resulting in the expression of EGFR target genes, including vascular endothelial growth factor (VEGF). Furthermore, EGFRvIII-containing EVs induce both morphological changes and anchorage-independent growth in recipient cells. Interestingly, EGFRvIII mRNA is also detectable in GBM-derived EVs [127]. Given that some reports demonstrated the heterogeneous distribution of EGFRvIII expression in GBM tissue [128, 129], the horizontal propagation of EGFRvIII by EVs might contribute to intratumoral heterogeneity and the progression of GBM. Such intracellular interactions by EVs between malignant tumor cells and less aggressive tumor cells were also observed in other types of cancer. Le et al. showed that highly metastatic breast cancer cells could transfer the EVs contained miR-200 family miRNAs to non-metastatic breast cancer cells to promote lung metastasis [130]. This miR-200 transfer induces the mesenchymal to epithelial transition (MET) by altering the expression of genes, including zeb2 and sec23a, in non-metastatic breast cancer cell lines. Qu et al. demonstrated that long non-coding RNAs (lncRNAs) were also transferred by EVs and contributed to the chemoresistance of renal cell carcinoma (RCC) cells [131]. This lncRNA, termed lncARSR, acts as a competitor of miR-34/miR-449 and stimulates AXL and c-MET expression to enhance chemoresistance in drug-sensitive RCC cells. From these reports, cancer-derived EVs play a crucial role in the acquisition and transfer of the malignant trait by horizontal propagation of oncogenic molecules.

**Table 5:** EV interaction between cancer cells and another cancer cells

Cell types of EV donor	Cell types of EV recipient	EV components	Functions	References
Positive regulation of extracellular vesicles on cancer progression				
Cancer cells	Cancer cells	H-ras and c-myc	Induce tumorigenic phenotypes	[12]
		EGFR variant III (EGFRvIII)	Induce both morphological changes and anchorage-independent growth	[126]
		EGFRvIII mRNA	Stimulate cancer cell proliferation	[127]
		Integrin	Promote adhesion and migration	[132]
		miR-200 family	Induce the mesenchymal to epithelial transition (MET) to promote metastasis	[130]
		Several miRNAs including miR-584	Activate JNK/p38 MAPK pathway and promote tumor growth	[133]
		miR-10b	Promte invasion activity	[134]
		Several miRNAs including miR-100, miR-222, and miR-30a	Increase survival rate	[135]
		miR-222	Promote invesion and motility	[136]
		Long non-coding RNA (lncARSR)	Stimulate AXL and c-MET expression to enhance chemoresistance	[131]

## Interaction between non-tumoral epithelial cells and cancer cells via EVs

Competitive cell interactions represent a basic biological process to maintain homeostasis [137]. In particular, during cancer initiation, aberrant cells bearing genetic or epigenetic mutations will conflict with the surrounding non-aberrant normal cells to eliminate them from the cell population [137, 138]. If these aberrant cells eliminate normal cells from tissue, it may lead to cancer formation and progression. Therefore, it is conceivable that normal cells require an eradication system for aberrant cells to prevent tumor initiation. Normal prostate epithelial cell lines secreted EVs to transfer the tumor suppressor miR-143 into cancer cells [85] (Table 6), resulting in the induction of growth inhibitory signals in prostate cancer cells. These results suggest that EVs may contribute to the maintenance of normal growth and prevent cancer initiation. In contrast, cancer cells also utilize EVs to overcome this cell competition with non-tumoral epithelial cells during the cancer initiation step. Breast cancer-derived EVs are capable of inducing anchorage-independent growth and survival in mammary epithelial cells [139] (Table 6). Similar to this report, high metastatic hepatocellular carcinoma cell lines also transfer the oncogenic molecules to the non-tumoral immortalized hepatocytes and promote invasive activity of the immortalized hepatocytes [140]. Therefore, cancer-derived EVs contribute to the expansion of cancer cells by transforming non-aberrant normal cells. From these reports, it is conceivable that EVs play an important role in cell competition between cancer cells and non-tumoral epithelial cells. Further investigation will advance our understanding of the progression mechanisms of competitive cellular interaction between cancer cells and epithelial cells.

**Table 6:** EV interaction between cancer cells and epithelial cells

Cell types of EV donor	Cell types of EV recipient	EV components	Functions	References
Positive regulation of extracellular vesicles on cancer progression				
Cancer cells	Epithelial cells	Tissue transglutaminase (tTG)	Induce anchorage-independent growth and survival	[139]
Epithelial cells	Cancer cells	miR-143	Induce growth inhibitory signals	[85]

## Conclusion

The precise mechanisms of intercellular communication in the tumor microenvironment remain obscure because there are many important pathways that modulate multiple non-EV factors, such as growth factors, cytokines, and chemokines. However, as described above, the rapid development of EV research elucidated the novel mechanism underlying the intrinsic intercellular communication networks during cancer initiation and progression. EVs possess impressively diverse functions in the intercellular communication networks in the tumor microenvironment. Cancer cells secrete EVs and dictate the phenotypes of surrounding cells to promote cancer progression. Against such “education,” non-tumoral cells utilized EVs to suppress cancer initiation and progression. Understanding the precise mechanisms of EVs in cancer biology may provide a breakthrough in the diagnostic and prognostic tools and therapeutic strategies of cancer.
